# Staged graft replacement with thoracic endovascular aneurysm repair for an extensive thoracoabdominal aortic aneurysm after total arch replacement

**DOI:** 10.1186/s13019-022-01764-3

**Published:** 2022-02-21

**Authors:** Kazufumi Yoshida, Ken Nakamura, Masanosuke Ishigami, Makoto Kinoshita, Tadaaki Koyama

**Affiliations:** 1 Department of Cardiovascular Surgery, Kobe City Medical Center General Hospital, 2-1-1 Minatojimaminamimachi Chuo-Ku, Kobe, 650-0047 Japan; 2 Department of Cardiovascular Medicine, Kobe City Medical Center General Hospital, Kobe, Japan

**Keywords:** Case series, Hybrid staged repair, Thoracoabdominal aneurysm, Total arch replacement, Thoracic endovascular aneurysm repair, Thoracoabdominal aortic replacement

## Abstract

**Background:**

Open surgery for thoracoabdominal aortic aneurysm is highly invasive. Staged repair for extensive TAAA is effective because it has low morbidity and mortality, and preserves spinal cord perfusion. An initial total arch replacement can create a proximal landing zone for thoracic endovascular aneurysm repair.

**Case presentation:**

We performed a staged hybrid thoracoabdominal aortic aneurysm repair after total arch replacement, which consisted of a primary open repair procedure as Crawford Extent III and IV thoracoabdominal aortic aneurysms, and a secondary thoracic endovascular aneurysm repair for the residual lesions for four patients. No spinal cord injury was observed. In one patient, the residual descending aortic aneurysm ruptured six months after the primary open surgery.

**Conclusions:**

Overall, staged hybrid repair is effective and shows low morbidity and mortality. Secondary thoracic endovascular aneurysm repair should be performed as soon as possible to reduce the risk of residual aneurysm rupture.

## Background

Open surgery for thoracoabdominal aortic aneurysm (TAAA) is highly invasive, especially for Crawford Extent II aneurysms, and it is associated with significant morbidity and mortality. Staged hybrid repair of TAAAs, which consists of thoracic endovascular aortic repair (TEVAR) of the proximal segment with a stent graft and open distal TAAA repair through a thoracoretroperitoneal incision, has been reported [1]. However, not all patients with Crawford Extent II TAAAs have adequate distal landing zones for TEVAR. To minimize the surgical stress on the TAAAs, we performed a staged hybrid repair for TAAAs in four patients, who underwent initial total arch replacement (TAR). A primary open repair was performed, followed by a secondary TEVAR of the descending aorta. The indication of the diameter of the aneurysm is over 55 mm or rapid dilatation of the diameter, and the diameter of the clamp site was under 60 mm. We reported the results, advantages, and disadvantages of our procedure.

## Case presentation

**Fig. 1:**
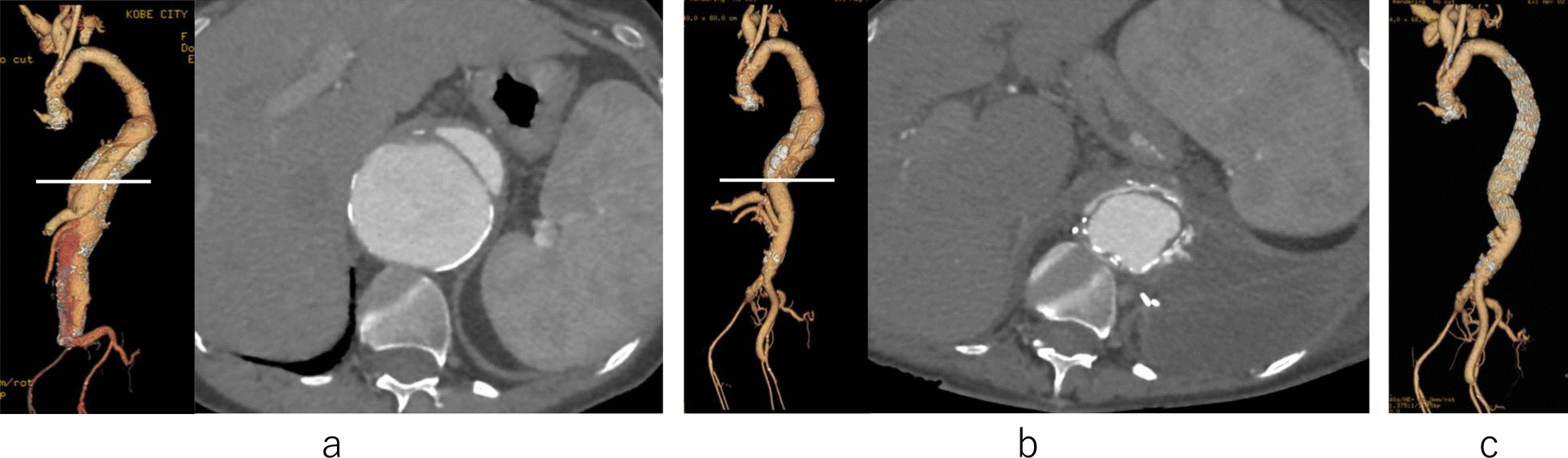
Three-dimensional computed tomography angiography (3D-CTA) images of case 1. **a** An axial cross-sectional image demonstrating the proximal anastomosis site (white line) in thoracoabdominal aortic replacement; **b** an axial cross-sectional image demonstrating the proximal anastomosis site (white line) after the primary open surgery. The peripheral anastomosis is the left external iliac artery; **c** the transabdominal aorta after hybrid repair

Case 1 involved a 44-year-old woman with Marfan syndrome who underwent Bentall surgery and mitral valve replacement 33 years ago. Total arch and descending aorta replacements were also performed with a 28-mm 4 Branch-Hemashiled Platinum Woven Double Velour graft (INTERVASCULAR SAS, LaCiotat, France) for a dissected thoracic aortic aneurysm 17 years ago. Computed tomography angiography (CTA) revealed an extensive dissected TAAA (Fig. 1a); the maximum diameters of the descending thoracic and abdominal aorta were 61 mm and 60 mm, respectively. The diameters of the bilateral external iliac arteries were < 6 mm each, and the Adamkiewicz artery could not be visualized. We scheduled a hybrid staged procedure with an initial open thoracoabdominal surgery followed by a TEVAR. Thoracoabdominal aortic replacement was performed using the following procedures: cerebrospinal fluid (CSF) drainage, perioperative monitoring of motor evoked potentials, partial extracorporeal circulation, selective visceral perfusion, and renal protection with cold lactated Ringer's solution. Mild hypothermia was instituted, with the minimum rectal temperature set at 34 °C. Proximal aortic clamping was performed at the descending aorta just above the diaphragm, and the tubular graft was anastomosed to the descending aorta with a double-barrel graft. The distal anastomosis was the left external iliac artery for the TEVAR. We used 30-mm Triplex 1 Branch (Terumo, Tokyo, Japan), 26-mm Coselli Thoracoabdominal (Terumo, Tokyo, Japan), and 22 mm × 11 mm Bifurcated Hemashield Platinum Woven Bifurcated Double Velour Vascular graft (INTERVASCULAR SAS) grafts (Fig. 1b). The patient underwent postoperative reintubation and tracheotomy owing to sputum excretion difficulty. Forty-eight days after the initial operation, TEVAR was performed using 32–200 mm and 38–200 mm Zenith TX stent grafts (Cook Medical, Indiana, United States). Postoperative CTA showed no abnormal findings, (Fig. 1c) and no spinal cord injury (SCI) was observed. The patient was weaned from mechanical ventilation and discharged on the 126th postoperative day. No aortic-related event occurred in long term. 54 months after the TEVAR, the patient was dead due to subarachnoid hemorrhage.

**Fig. 2:**
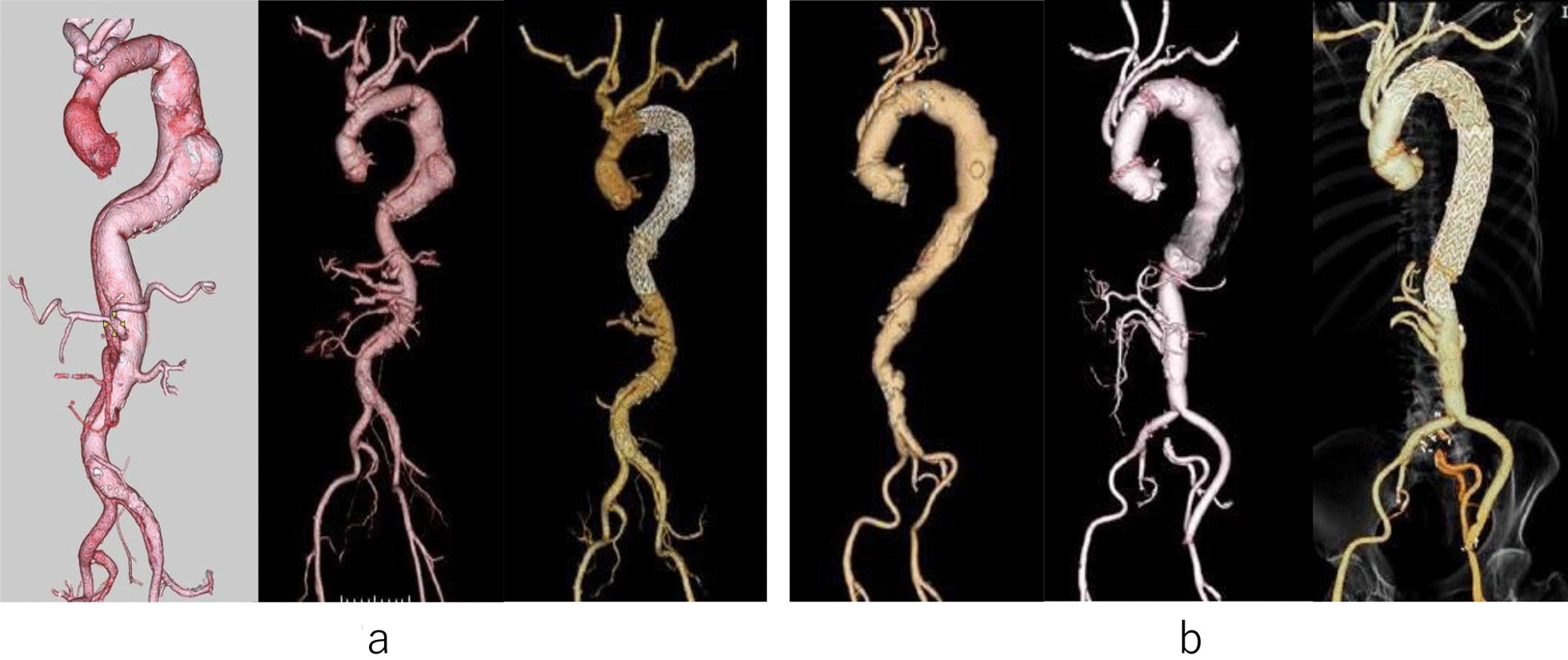
3D-CTA images of case 2 and 3. **a** The abdominal aorta of case 2 during the preoperative period and after the primary open surgery and hybrid repair; **b** the abdominal aorta of case 3 during the preoperative period and after the primary open surgery and hybrid repair

Case 2 involved a 65-year-old man who underwent TAR and proximal descending aorta replacement with a 26-mm UBE woven graft (Ube Industries, Tokyo, Japan) for type A aortic dissection 12 years ago. CTA revealed an extensive dissected TAAA; the maximum diameters of the descending thoracic and abdominal aortas were 55 mm and 45 mm, respectively. The Adamkiewicz artery was visualized as arising from the 12th intercostal artery. Thoracoabdominal replacement was performed similarly to that performed for the first patient. The descending aorta and Adamkiewicz artery were reconstructed using double-barrel anastomosis and two tubular grafts (24-mm thoracoabdominal J graft), respectively. TEVAR was subsequently performed with 31–200-mm and 31–200-mm Gore CTAG stent grafts (WL Gore, Newark, Delaware, United States) 19 days after the initial operation. No abnormal finding was observed with the postoperative CTA (Fig. 2a), and no SCI was occurred. The patient was discharged on the 34th postoperative day with no complications. Embolization of type 2 endoleak was performed 38 months after the TEVAR, and the patient are alive 44 months after the operation.

Case 3 involved an 81-year-old woman who underwent TAR with a 28-mm Triplex 4 Branch graft (Terumo, Tokyo, Japan) for an aortic arch aneurysm three months ago. The patient complained of back pain, and the CTA demonstrated a type B aortic dissection with an ulcer-like projection (ULP). The follow-up CTA indicated rapid dilatation of the ULP. The maximum diameters of the descending thoracic and abdominal aortas were 52 mm and 51 mm, respectively. The Adamkiewicz artery was not visualized. The thoracoabdominal aortic aneurysm was replaced with 28-mm thoracoabdominal J and 20 mm × 11 mm Hemashield Platinum Woven Bifurcated Double Velour Vascular graft (INTERVASCULAR SAS). Paraparesis was observed on the first postoperative day, but the patient recovered fully. No neurologic deficits were observed after the CSF drainage, and a mean blood pressure of > 85 mmHg was attained. TEVAR was performed with 31–200-mm Gore CTAG and 36–36-250-mm Relay plus (Bolton Medical, Sunrise, Florida, United States) grafts 30 days after the primary operation. The postoperative CT showed excellent graft patency without major endoleaks (Fig. 2b). The patient was transferred to the rehabilitation center on the 68th postoperative day. No aortic-related event occurred in long term. The patient are alive and 45 months after the operation.

**Fig. 3:**
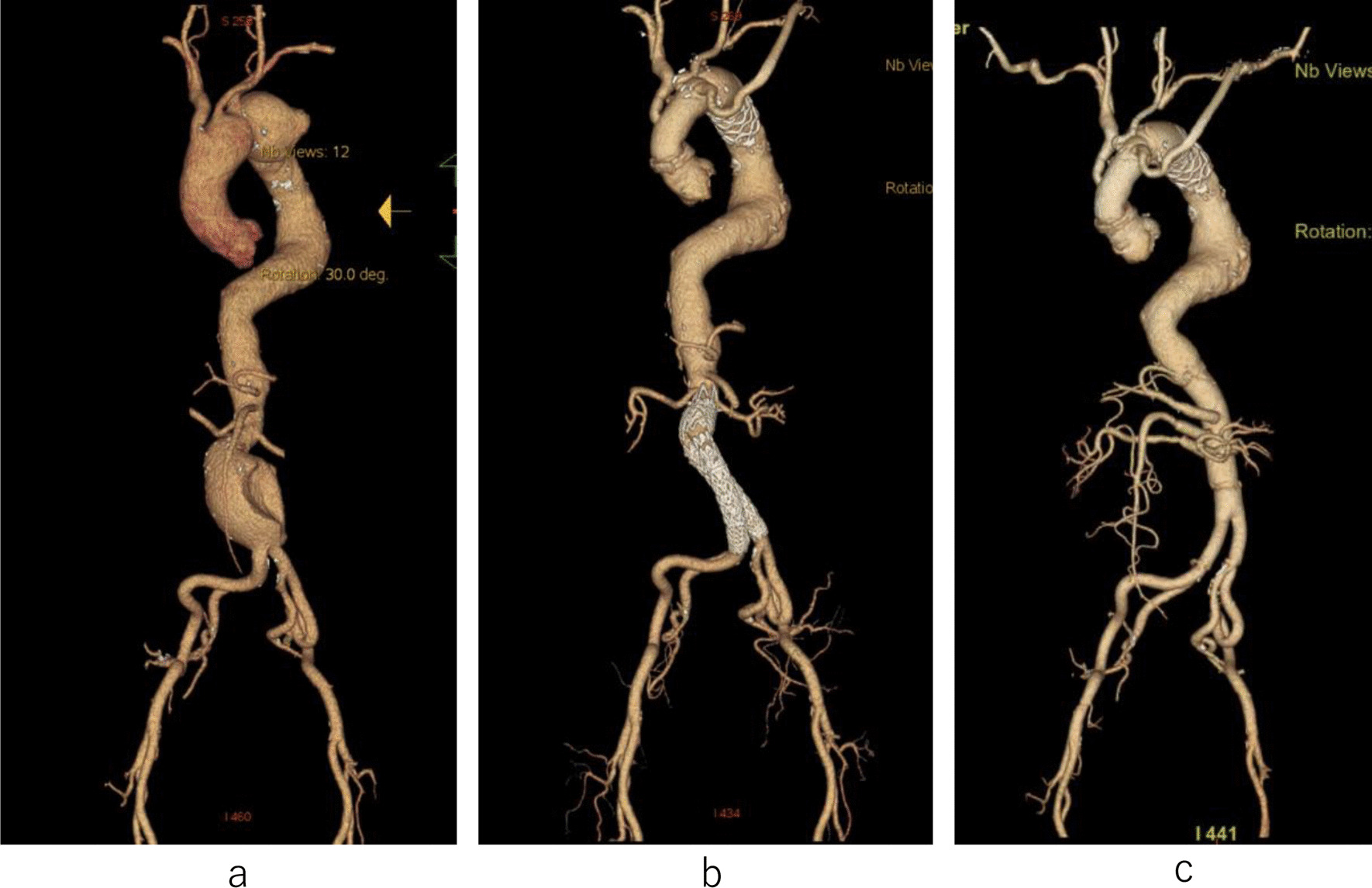
3D-CTA images of case 4. **a** The thoracic and abdominal aortic aneurysms in case 4; **b** The abdominal aorta after endovascular aneurysm repair and total arch repair with the frozen elephant trunk procedure; **c** the dilated descending aortic aneurysm after thoracoabdominal replacement

Case 4 involved a 75-year-old man who underwent abdominal endovascular aortic repair (EVAR) for an abdominal aortic aneurysm. One month later, TAR with a frozen elephant trunk was performed with 26-mm Triplex 4 Branch (Terumo) and 35–60-mm J FROZENIX (Japan Lifeline, Tokyo, Japan) grafts for an aortic arch aneurysm (Fig. 3a, b). Eighteen months later, the patient complained of fever, and CT showed increased fluid around the stent graft, which indicated that the aneurysm had gradually dilated. The Adamkiewicz artery was visualized as arising from the 10th intercostal artery. A thoracoabdominal graft replacement was performed for the stent graft infection. The infected stent graft was explanted and the descending aorta was reconstructed at the level of the 12th intercostal artery (Fig. 2c). Anaphylactic shock from the contrast media occurred when the postoperative CT was performed. Considering the risk for SCI and anaphylaxis, we decided to monitor the descending aortic aneurysm closely. Six months later, the aneurysm ruptured, and we performed emergent TEVAR. However, acidemia and hyperpotassemia progressed, and the patient died on the same day.

## Discussion

We performed a staged hybrid TAAA repair, which consisted of a primary open repair procedure as Crawford Extent III and IV TAAAs, and a secondary TEVAR for the residual lesions for four patients. Open repair of extensive Crawford Extent I and II TAAA is associated with a high mortality rate of 7.4–11.1% [2–4]. When performed after TAR, open surgery for Crawford Extent I and II TAAA may be difficult because of adhesions between the lung and descending aorta. Amit et al. described a hybrid repair approach that can treat extensive TAAAs by performing an initial TEVAR to convert Crawford Extent I and II TAAAs into Crawford Extent III or IV TAAAs and a subsequent open repair to treat the residual lesions [1, 5]. Etz et al. reported a 15% and 0% incidence of paraplegia in the single-stage and two-stage open repairs, respectively [4] because the staged strategy allows the spine to develop a collateral network. Staged repair for extensive TAAA is effective because it has low morbidity and mortality, preserves spinal cord perfusion, and prevents lung injury.

Several methods for the staged repair of TAAAs have been reported. Amit et al. initially performed endovascular repair of the proximal aortic segment with a thoracic stent graft followed by an open distal TAAA repair through a thoracoretroperitoneal incision [1]. Kihara et al. performed primary Y-grafting with the debranching technique, secondary TAR, and TEVAR between the proximal and distal landing zones [6]. In this study, TEVAR could not be initially performed because of the inadequate distal landing zone, so TAAA repair was performed first as Crawford IV TAAAs and a secondary TEVAR was followed for residual lesions. Staged hybrid repair can allow to avoid the perioperative complications (such as SCI, recurrent nerve injury, lung injury). Additionally, compared to previous studies, our method has four advantages. First, we created distal landing zones, which was important because some patients with Crawford Extent II TAAA do not have adequate distal landing zones for TEVAR. TAAA replacement as Crawford Extent III and IV TAAAs also makes it easier to perform TEVAR because of the suitable proximal and distal landing zones. Second, if the access routes are still inadequate for TEVAR, our technique allows the leg of the graft to be extended towards the external iliac artery for the secondary TEVAR. Third, this treatment strategy can be accomplished with a combination of simple techniques. our strategy combines an open repair approach with TEVAR and is superior to the Y-grafting with the complicated debranching technique. Fourth, even if a SCI occurs, After TAVER, bleeding and coagulation abnormalities do not occur and spinal cord protection strategies can be performed more safely. it is often difficult to control blood pressure after thoracoabdominal replacement because of bleeding and coagulation abnormalities. Our method has one disadvantage. This approach requires proximal clamping and anastomosis of the descending aorta, which cannot be performed in some cases, particularly among patients with mega or shaggy aortas.

The appropriate period between thoracoabdominal replacement and TEVAR remains controversial. Kihara et al. performed TEVAR 50 days after the initial Y-grafting with the debranching technique [6]. In a study by Gombert et al. both stages were performed with an interval of 14.2 ± 15.3 days (n = 6) [7]. Complications from the primary open surgery in case 1 caused a delay in the secondary TEVAR; however, secondary TEVAR should be performed as soon as possible because of the risk for rupture of the residual aneurysm. In case 4, the aneurysm ruptured six months after the open surgery. TEVAR should be performed within 7–30 days after thoracoabdominal replacement, which provides enough time for the development of a spinal perfusion collateral network with the least risk for aneurysm rupture.

For cases of TAAA that extend to the aortic arch, an initial TAR with an elephant trunk procedure should be performed, followed by a hybrid repair using our strategy. This option should be considered for patients with an enlarged aortic arch that is difficult to dissect via thoracoretroperitoneal exposure.

## Conclusion

Our study demonstrated that patients with extent TAAA who underwent TAR can benefit from staged hybrid repair, which consists of an initial open repair as Crawford Extent III and IV TAAAs, and secondary TEVAR for residual lesions. This strategy is an effective method for the preservation of spinal cord perfusion, and it shows low morbidity and mortality.

## Data Availability

All data generated or analyzed are included in this article.

## Abbreviations

TAAA: Thoracoabdominal aortic aneurysms
TAR: Total arch replacement
TEVAR: Thoracic endovascular aneurysm repair
CTA: Computed tomography angiography
CSF: Cerebrospinal fluid
SCI: Spinal cord injury
ULP: Ulcer-like projection
EVAR: Abdominal endovascular aortic repair

## References

[1] Jain A, Flohr TF, Johnston WF, Tracci MC, Cherry KJ, Upchurch GR (2016). Staged hybrid repair of extensive thoracoabdominal aortic aneurysms secondary to chronic aortic dissection. J Vasc Surg.

[2] Coselli JS, LeMaire SA, Preventza O, de la Cruz KI, Cooley DA, Price MD (2016). Outcomes of 3309 thoracoabdominal aortic aneurysm repairs. J Thorac Cardiovasc Surg.

[3] Cambria RP, Clouse WD, Davison JK, Dunn PF, Corey M, Dorer D (2002). Thoracoabdominal aneurysm repair: results with 337 operations performed over a 15-year interval. Ann Surg..

[4] Etz CD, Zoli S, Mueller CS, Bodian CA, Di Luozzo G, Lazala R (2010). Staged repair significantly reduces paraplegia rate after extensive thoracoabdominal aortic aneurysm repair. J Thorac Cardiovasc Surg.

[5] Johnston WF, Upchurch GR, Tracci MC, Cherry KJ, Ailawadi G, Kern JA (2012). Staged hybrid approach using proximal thoracic endovascular aneurysm repair and distal open repair for the treatment of extensive thoracoabdominal aortic aneurysms. J Vasc Surg.

[6] Kihara K, Tamura K, Chikazawa G, Sakaguchi T, Totsugawa T, Yoshitaka H (2015). Hybrid three-stage repair for extended thoracoabdominal aortic aneurysm: report of a case. Ann Vasc Dis.

[7] Gombert A, Kirner L, Ketting S, Rückbeil MV, Mees B, Barbati ME (2019). Editor’s choice—outcomes after one Stage versus two stage open repair of Type II thoraco-abdominal aortic aneurysms. Eur J Vasc Endovasc Surg.

